# The role of nutritional support in the physical and functional recovery of critically ill patients: a narrative review

**DOI:** 10.1186/s13054-017-1810-2

**Published:** 2017-08-26

**Authors:** Danielle E. Bear, Liesl Wandrag, Judith L. Merriweather, Bronwen Connolly, Nicholas Hart, Michael P. W. Grocott, on behalf of the Enhanced Recovery After Critical Illness Programme Group (ERACIP) investigators

**Affiliations:** 1 Department of Nutrition and Dietetics, Guy’s and St Thomas’ NHS Foundation Trust, London, UK; 2 Department of Critical Care, Guy’s and St Thomas’ NHS Foundation Trust, London, UK; 3 Division of Asthma, Allergy, and Lung Biology, King’s College London, London, UK; 4 National Institute for Health Research (NIHR), Guy’s and St Thomas’ NHS Foundation Biomedical Research Centre, London, UK; 5 Lane Fox Clinical Respiratory Physiology Research Centre, Guy’s and St Thomas’ NHS Foundation Trust, London, UK; 6 Anaesthesia, Critical Care and Pain Medicine, University of Edinburgh, Royal Infirmary of Edinburgh, Edinburgh, UK; 7 Centre for Human and Aerospace Physiological Sciences, King’s College London, London, UK; 8 Department of Physiotherapy, The University of Melbourne, Melbourne, VIC Australia; 9 Integrative Physiology and Critical Illness Group, Clinical and Experimental Sciences, University of Southampton, Southampton, UK; 10 Respiratory and Critical Care Research Theme, Southampton NIHR Biomedical Research Centre, Southampton, UK; 11 Anaesthesia and Critical Care Research Unit, University Hospital Southampton NHS Foundation Trust, Southampton, UK

**Keywords:** Nutrition, Critical illness, Physical recovery, Energy, Protein

## Abstract

The lack of benefit from randomised controlled trials has resulted in significant controversy regarding the role of nutrition during critical illness in terms of long-term recovery and outcome. Although methodological caveats with a failure to adequately appreciate biological mechanisms may explain these disappointing results, it must be acknowledged that nutritional support during early critical illness, when considered alone, may have limited long-term functional impact.

This narrative review focuses specifically on recent clinical trials and evaluates the impact of nutrition during critical illness on long-term physical and functional recovery.

Specific focus on the trial design and methodological limitations has been considered in detail. Limitations include delivery of caloric and protein targets, patient heterogeneity, short duration of intervention, inappropriate clinical outcomes and a disregard for baseline nutritional status and nutritional intake in the post-ICU period.

With survivorship at the forefront of critical care research, it is imperative that nutrition studies carefully consider biological mechanisms and trial design because these factors can strongly influence outcomes, in particular long-term physical and functional outcome. Failure to do so may lead to inconclusive clinical trials and consequent rejection of the potentially beneficial effects of nutrition interventions during critical illness.

## Background

Many basic questions about the provision of nutritional support to critically ill patients remain unanswered [1]. Outcome from critical illness has previously been measured using relatively blunt outcome measure tools such as mortality, days on mechanical ventilation and rates of acquired infection. Recent randomised controlled trials (RCTs) have not shown any mortality benefit when specific nutritional interventions have been investigated over the first week of critical illness [2–4] and other studies have reported harm [5–7]. This has led to useful debate regarding the most appropriate timing, type and amount of nutrition support that should be delivered to critically ill patients.

It is generally accepted that providing some enteral nutrition (EN) early (within 48–72 hours of admission) modulates the immune response and reduces oxidative stress and infections by limiting bacterial translocation via the gut [1, 8, 9]. In addition, provision of nutrition over the course of critical illness may alter the composition and function of the host microbiome [10], further influencing the immune response. However, the clinical impact of this physiological observation is unknown. In contrast, it has been hypothesised that early feeding blunts autophagy, preventing adequate clearance of damaged cells and resulting in muscle wasting and increased muscle weakness [1, 7].

As the number of patients surviving critical illness has risen, there has been an increase in reported physical and functional disability as well as impairment of quality of life following discharge from the intensive care unit (ICU) [11–13]. Body composition and physical and functional impairment have been measured using a variety of tools at different time points over the course of critical illness and the patient’s recovery trajectory [14, 15]. At least 33 different measures of skeletal muscle mass, strength and function have been identified for use in critically ill patients [14]. These measures include both volitional and non-volitional tools, with varying reliability and validity. Currently, the most appropriate measure to use at each time point and for each intervention is unknown. Whichever measure is used, it is clear that recovery in survivors from critical illness is poor, even up to 5 years post discharge from the ICU [13].

Skeletal muscle weakness, termed intensive care unit-acquired weakness (ICU-AW), contributes significantly to the physical and functional disability observed in these patients. Skeletal muscle wasting, both early in critical illness [16] and potentially ongoing as a result of persistent inflammatory catabolic syndrome (PICS) [17], has been identified as a contributing factor to ICU-AW [18]. Furthermore, low skeletal muscle mass on admission to the ICU has been shown to be a predictor of poor outcome [19]. It is here, in the reduction of skeletal muscle wasting and the recovery of survivors of critical illness, that nutrition support may prove the most beneficial, but there is remarkably little nutrition research specifically focusing on these outcomes.

The aims of early nutrition support in critically ill patients are often cited as the reduction of catabolism, attenuation of muscle wasting and maintenance of nutritional status [8, 9]. However, to date there has been limited focus on muscle wasting and functional performance as outcomes in critical care nutrition trials. This narrative review will discuss clinical trials which have evaluated the physical and functional impact of critical care nutrition interventions, either as secondary outcomes, sub-studies or post-hoc analyses, as well as the effect of timing, type and amount of nutrition support on recovery following critical illness. Particular attention will be given to factors that should be considered in the design of future RCTs of nutrition in this patient group, which will probably benefit from adoption of a translational science approach.

## Timing of nutrition support

Most experts and guidelines agree that EN should be commenced within 24–48 hours of admission to the ICU [1, 8, 20, 21]. Early EN is encouraged to assist with the maintenance of gut integrity, modulation of the stress and immune response and attenuation of disease severity [22, 23], which may, in turn, improve overall outcome [24]. The most recent meta-analysis of trials investigating the effect of early EN was performed as part of the joint American Society for Parenteral Nutrition (ASPEN) and Society of Critical Care Medicine (SCCM) guidelines for the provision of nutrition support in critical illness [8]. This systematic review identified 21 RCTs meeting their inclusion criteria and found that provision of early EN was associated with a significant reduction in mortality and infectious morbidity compared with withholding early EN (delayed EN or standard care) [8].

In contrast to this convincing evidence, others argue that anorexia may be a preserved evolutionary response and that early starvation or limiting nutritional intake over the first 48–72 hours to the first week of critical illness is beneficial [25, 26]. This notion contrasts with results of many observational studies reporting that feeding via the enteral route alone leads to significant underfeeding which in turn is negatively associated with the standard outcome measures of mortality, length of stay and infection frequency [27–30]. As a consequence of these findings, the use of PN has increased over recent years, which raises further questions in relation to the timing of nutrition support.

In the large EPaNIC trial (Early Parenteral Nutrition Completing Enteral Nutrition in Adult Critically Ill Patients) [7], the use of early PN to supplement early insufficient EN led to greater muscle weakness, thought to be related to impaired autophagy [31]. For this reason, the use of PN (either exclusive or supplemental) is not recommended over the first 7 days of ICU admission in patients who are not considered to be at high nutritional risk [8].

Despite these opposing views, studies investigating the impact of withholding nutrition completely over the first week of critical illness do not exist and guidelines continue to recommend increasing nutrition support over the first week of critical illness to meet target recommendations [8, 20, 21]. Additionally, studies undertaken after the first week of critical illness, and indeed in the post-ICU phase, are lacking. This will be discussed later in the review, but essentially the impact of usual nutritional practice in the ICU on the physical and functional recovery of ICU patients is unknown.

The presumed benefit of nutritional support during critical illness, in order to reduce muscle wasting, is based on three assumptions. The first assumption is that all patients absorb all of the nutrients delivered; the second is that the critically ill skeletal muscle can utilise the nutrients which are delivered; and the third assumption is that the consequence of these processes is always an anabolic and never a catabolic effect [32]. Contrary to these assumptions, delays in gastric emptying [33] and incomplete absorption from the small bowel [34] may significantly alter the presumed benefit. In addition, little is known about the ability of skeletal muscle to utilise these nutrients at different time points over the ICU admission. It is possible that current feeding methods may not physiologically be able to produce the desired outcome benefit or that provision of nutrients does not result in anabolism, particularly in the earliest phase of critical illness (e.g. first 48–72 hours) [35] or in clinical conditions defined by persistent inflammation and hypoxia [16]. Studies investigating the anabolic effect of nutrition at different time points over the course of critical illness and recovery are required to provide further guidance on the most appropriate timing of nutrition support in order to influence these outcomes.

## Dose of nutrition support

### Energy

In general, critically ill patients do not meet recommended levels of nutritional intake, particularly when the enteral route is used alone [36]. This is true both in routine clinical practice [36] and in the setting of RCTs [2–4]. The effect of underfeeding during the period of critical illness on skeletal muscle wasting and physical function is wholly unclear. One-year follow-up from the EDEN trial (Early vs Delayed Enteral Feeding to Treat People with Acute Lung Injury or Acute Respiratory Distress Syndrome) suggested that there was no beneficial effect on physical function from target compared with trophic enteral feeding over the first 6 days of critical illness [37], albeit in the context of a number of confounders that would need further consideration. However, more patients in the trophic feeding group were discharged to rehabilitation centres, suggesting that there may be some beneficial effect to improving nutritional intakes [37]. Noteworthy in this trial is that patients in the full feeding group only met 70% of the energy targets which may not be sufficient to produce an outcome benefit, at least when predictive equations are used [30]. In contrast, a sub-group analysis from the Reducing Deaths due to Oxidative Stress Study (REDOXs) found that increasing nutritional adequacy led to improvements in 3-month Short Form-36 (SF-36) scores relating to the physical domains. However, this effect was diminished by 6 months [38]. Other large RCTs have also included physical or quality of life outcomes with varying results (Table 1) [2, 3, 31, 38–42].

**Table 1:** Randomised controlled trials of nutrition in critically ill patients reporting physical and functional outcomes

	NHLBI et al., 2012 [3]	Needham et al., 2013 [37]	Casaer et al., 2013 [39]	Hermans et al., 2013^a^ [31]	Doig et al., 2013 [40]	Harvey et al., 2014 [2]	Wei et al., 2015 [38]	Doig et al., 2015 [41]	Ferrie et al., 2016 [42]
Patient population	*N* = 1000 ALI	*N* = 174 ALI from EDEN cohort	*N* = 15 Neurosurgical patients from EPaNIC cohort	*N* = 600 30% cardiac surgery	*N* = 1372 45.85% emergency surgery 19.9% elective surgery	*N* = 2400 13% surgical	*N* = 475 78% medical	*N* = 339 64.5% medical 24% emergency surgical 12% elective surgical	*N* = 129 Requiring PN
Study design	Multicentre, 44-site RCT	Multicentre, 5-site follow-up from EDEN	Prospective sub-study of large RCT	Sub-study from EPaNIC	Multicentre, 31-site RCT	Multicentre, 33-site pragmatic RCT	Retrospective analysis from REDOXs	Multicentre, 13-site RCT	Single-centre RCT
Duration of intervention	6 days	6 days	9 days	10 days	NR	5 days (120 hours)	8 days	7 days	Up to 10 days
Age, years (mean)	Trophic feeding: 52 Full feeding: 52	Trophic feeding: 48 Full feeding: 47	Early PN: 44 Late PN: 50	Early PN: 62 Late PN: 65 (median)	Standard care: 68.6 Early PN: 68.4	PN: 63.3 EN: 62.9	Low nutritional adequacy: 62 Moderate nutritional adequacy: 62 High nutritional adequacy: 65	Standard: 61 Restricted: 59	0.8 g/kg amino acids: 64.5 1.2 g/kg amino acids: 67.0
BMI	Trophic feeding: 29.9 Full feeding: 30.4	Trophic feeding: 31 Full feeding: 32	Early PN: 24 Late PN: 25	Early PN: 25 Late PN: 24.9 (median)	Standard care: 28.5 Early PN: 27.9	PN: 27.7 EN: 28.2	Low nutritional adequacy: 29.7 Moderate nutritional adequacy: 30.4 High nutritional adequacy: 29.3	Standard: 28 Restricted: 28	0.8 g/kg amino acids: 27.4 1.2 g/kg amino acids: 25.7
APACHE II score	NR	NR	Early PN: 28 Late PN: 30	Early PN: 32 Late PN: 30 (median)	Standard care: 21.5 Early PN: 20.5	PN: 15.1 EN: 15.2	Low nutritional adequacy: 27.2 Moderate nutritional adequacy: 26.8 High nutritional adequacy: 26.6	Standard: 18 Restricted: 18	0.8 g/kg amino acids: 23.7 1.2 g/kg amino acids: 25.5
Mortality^b^ (%)	Trophic feeding: 23.2 (60 days) Full feeding: 22.2 (60 days)	Trophic feeding: 2 Full feeding: 7 (before 6-month follow-up)	Early PN: 20 Late PN: 0 (90 days)	Early PN: 13 Late PN: 10	Standard care: 14.66 Early PN: 11.89	PN: 26.6 EN: 29.4	Low nutritional adequacy: 26 Moderate nutritional adequacy: 27 High nutritional adequacy: 23	Standard: 9 Restricted: 5	0.8 g/kg amino acids: 6 1.2 g/kg amino acids: 8
LOS (ICU)^c^	NR	Trophic feeding: 15.8 Full feeding:13.4	Early PN: 12 Late PN: 9	Early PN: 11 Late PN: 13	Standard care: 9.3 (mean) Early PN: 8.2 (mean)	PN: 8.1 days EN: 7.3 days	Low nutritional adequacy: 18 Moderate nutritional adequacy: 19 High nutritional adequacy: 18	Standard: 10.0 (mean) Restricted: 11.4 (mean)	0.8 g/kg amino acids: 6.0 1.2 g/kg amino acids: 5.0
Provision of energy	Trophic feeding: 400 kcal/day Full feeding: 1300 kcal/day	NR	Early PN: approximately 21 kcal/kg Late PN: approximately 14 kcal/kg (over period of CT scans)	Overall total NR Early PN: < 30 kcal/kg/day Late PN: < 25 kcal/kg/day	Overall total not reported, but < 1600 kcal/day	PN: 21 kcal/kg/day EN: 18.5 kcal/kg/day	Mean for each group NR, but overall 56%	Overall total NR Standard: < 1600 kcal/day each day Restricted: < 1400 kcal/day each day	0.8 g/kg amino acids: 24.9 kcal/kg 1.2 g/kg amino acids: 23.1 kcal/kg (first 7 days only)
Provision of protein	NR	NR	NR	Overall total NR Early PN < 1.0 g/kg/day Late PN: < 0.9 g/kg/day	Total not reported, but 60 g/day	PN: 0.7 g/kg/day EN: 0.6 g/kg/day	Mean for each group NR, but overall 51%	Overall total NR Standard: < 60 g/day each day Restricted: < 55 g/day each day	0.8 g/kg amino acids: 0.9 g/kg 1.2 g/kg amino acids: 1.09 g/kg
Outcomes	↑ number of patients discharged to rehabilitation facilities in trophic feeding group	No difference in physical function at 1 year	Early PN ↓ femoral muscle quality (increased intramuscular water/lipid content) at median of ICU day 9	↓ weakness on first assessment (median day 9) Faster recovery of weakness Favouring late PN	Improved SGA scores per week (↓ muscle wasting and ↓ fat loss) ↑ RAND-36 scores at 60 days All favouring early PN	No difference in HRQoL at 1 year using EQ-5D-5 L	Improved functional aspects of HRQoL at 3 months with improved nutritional adequacy	↓ general health at day 90 in restricted caloric group using RAND- 36 survey	↑ handgrip strength at day 7 ↓ muscle loss using muscle ultrasound at day 7 ↓ Chalder fatigue score at day 7 favouring high amino acid group

*NHLBI* National Heart Lung and Blood Institute Acute Respiratory Distress Syndrome Clinical Trials Network, *ALI* acute lung injury, *NR* not reported, *LOS* length of stay, *BMI* body mass index, *EDEN* Early vs. Delayed Enteral Feeding to Treat People with Acute Lung Injury of Acute Respiratory Distress Syndrome, *EPaNIC* Early Parenteral Completing Enteral Nutrition in Adult Critically ill Patients, EQ-5D EuroQol 5 Dimension Questionnaire, *REDOXs* Reducing Deaths Due to Oxidative Stress Study, *SGA* subjective global assessment, *PN* parenteral nutrition, *EN* enteral nutrition, *ICU* intensive care unit, *HRQoL* health-related quality of life, *RCT* randomised controlled trial, *APACHE* Acute Physiology and Chronic Health Evaluation, *CT* computed tomography, *↑* increase, *↓* decrease

^a^Only data for patients assessed with Medical Research Council sum score presented here

^b^ICU mortality unless specified

^c^Median unless specified

Two pre-planned sub-group analyses from the EPaNIC study investigated the impact of the macronutrient dose (in the form of early vs late supplemental PN) on rates of skeletal muscle wasting [31, 39]. The first of these [31] found that muscle wasting, measured from muscle biopsies, was not different between the two groups. In addition, using the Medical Research Council (MRC) sum-score, weakness was found to recover faster in the group receiving late PN. In the second of these sub-group analyses [39], early PN was shown to adversely impact on femoral muscle quality, measured using computed tomography (CT) scans, but did not affect the rates of wasting observed in 15 neurosurgical patients.

It is likely that the timing and dose of energy provision go hand in hand. Indeed, recent thinking suggests that consideration of the endogenous production of energy in early critical illness is essential to the timing and dose of nutritional supplementation [1]. However, with no bedside method to measure endogenous energy production, it is impossible to account for this when calculating energy expenditure and devising feeding regimens. It has been postulated that in early critical illness (e.g. within the first 72–96 hours) permissive underfeeding to approximately 15 kcal/kg with full protein nutrition support may be warranted [43], but this awaits confirmation of benefit in RCTs. In addition, the use of predictive equations to determine energy targets may heavily influence the results of nutrition trials in the ICU as they are known to produce results which are less accurate than measured energy expenditure (MEE) using indirect calorimetry [44]. Indeed, studies feeding to MEE have consistently shown positive benefits and a recent observational study found that feeding to 70% of MEE was optimal in terms of mortality [45]. However, limitations preclude the frequent use of indirect calorimetry in clinical practice. These include availability of accurate metabolic monitors, costs, time taken to undertake the measurement and specific exclusions meaning that some of the sickest patients are unsuitable for measurement (e.g. those on continuous renal replacement therapy and those with high oxygen requirements) [46]. However, the introduction to the market of a metabolic monitor designed specifically for mechanically ventilated patients, with a reasonable cost, is under development and may bypass some of these limitations for future trials [46]. This is particularly pertinent as the effect of this targeted energy feeding on physical and functional recovery remains unknown.

### Protein

Inadequate protein provision has been considered a contributing factor explaining why RCTs, such as the EDEN trial mentioned earlier [3], do not show any beneficial impact of nutrition in the critically ill [47]. Early studies investigating protein intake in critically ill patients reported an improvement in whole-body nitrogen balance or whole-body protein turnover, with higher protein intakes [48]. Since then, several large observational studies have reported mortality benefits when higher protein delivery is achieved [49–51]. Whilst this may, in part, be because less sick patients may be able to have more protein delivered, this important confounder is accounted for in many of the more recent studies. For this reason, current recommendations range between 1.2 and 2.5 g/kg/day [8]. Whilst it seems plausible that higher protein delivery may attenuate skeletal muscle loss, the data supporting enhancement of muscle strength and function are lacking [52]. Secondary outcome results relating to the physical function component of the SF-36 score from the Nephro-Protective Trial [53], investigating the effect of intravenous amino acid supplementation on development of acute kidney injury, are awaited to contribute to the current evidence base.

One recent RCT investigated the effect of different protein intakes on muscle strength, wasting and fatigue in critically ill patients receiving PN [42]. In this study, 119 patients were randomised to receive 0.8 or 1.2 g/kg protein. There was no difference in the primary outcome of handgrip strength at ICU discharge. However, despite a smaller than planned difference in the delivery of protein (0.9 g/kg vs 1.1 g/kg), the study found that a higher protein intake resulted in differences in secondary outcomes including greater handgrip strength at day 7, improved measures of forearm muscle thickness and rectus femoris cross-sectional area and reduced fatigue scores. These results support the concept that a higher protein intake, at least when supplied via the parenteral route, leads to a reduction in muscle wasting during the first week of critical illness. However, such preliminary findings await confirmation in larger studies that would, in particular, need to correct for baseline heterogeneity, as these results are in contrast to observational data from the EPaNIC Study [31, 39] and from the MUSCLE-UK group where higher protein delivery was observed to be associated with greater skeletal muscle wasting [16].

Taken together, these data have led to the hypothesis that it may not be the amount of protein delivered, but the way in which we deliver the feed in a continuous manner that drives skeletal muscle wasting [32]. In healthy subjects, muscle protein synthesis increases from 45 to 90 minutes after provision of amino acids, either oral or intravenous, but then decreases after 90 minutes [54, 55]. This effect is observed despite the continued availability of amino acids in both the plasma and muscle, and has been termed the ‘muscle full effect’. It is not unreasonable to consider that this effect is also relevant in critically ill patients, and this hypothesis underpins the rationale for the current multi-centre RCT comparing intermittent and continuous feeding to investigate the effect on skeletal muscle wasting [35].

## Methodological challenges

### Patient selection

One of the major challenges in demonstrating benefit from nutrition in critically ill patients is the heterogeneous nature of the clinical population. In this regard it is unlikely that all patients will benefit from the same treatment at the same time point. Whilst feeding protocols are recommended as a means of encouraging early enteral feeding, a ‘one size fits all’ approach to the treatment of critically ill patients is no longer considered appropriate. However, defining patients who are likely to benefit from a nutritional intervention will be challenging and will require rigorous investigation. One method currently suggested to distinguish those patients who may benefit from a targeted nutrition intervention from those who may not is the determination of nutrition risk. The most popular nutritional risk scoring systems for critically ill patients are the Nutrition Risk Score 2002 (NRS 2002) [56] and the Nutrition Risk in the Critically Ill (NUTRIC) score [57, 58]. Although recent clinical trials have used the NRS 2002 in their inclusion criteria, this score may lack the specificity to determine the true nutrition risk of critically ill patients as an APACHE II score > 10 automatically gives the highest risk score. Although the NUTRIC score was developed specifically for critically ill patients, it is yet to be validated prospectively. However, post-hoc analyses from the PermiT (The Permissive Underfeeding versus target Enteral Feeding in Adult Critically Ill Patients) trial did not show a mortality improvement when stratifying by this score [59]. In addition, these scoring systems do not include variables relating to muscle mass or baseline physical function therefore more work is required in the area of nutrition risk before it can be used as a criterion in future nutrition studies.

Potential tools for patient selection in clinical trials include those to determine both baseline skeletal muscle quantity and quality and change over time. Such tools include muscle ultrasound and CT. However, there are limitations with the use of these tools outside the research setting. First, there is currently no standardised, universal technique to perform muscle ultrasound [60]; and second, only patients who have required a CT scan for clinical purposes have been investigated, meaning that a selection bias may be present [61]. Nonetheless, with further work to standardise techniques, these measures may prove useful in the future either on their own or as an adjunct to existing nutrition risk tools. Lastly, if physical or functional outcomes are to be included as important clinical outcomes, then the limitations surrounding obtaining baseline measurements for non-elective ICU admissions will be equally as important and further work needs to be undertaken to understand this in detail.

### Delivery of the intervention

Ensuring adequate delivery of the intervention is an essential factor in being able to interpret the outcome of such studies. Several studies in critical care nutrition have not been successful in reaching either target energy or protein delivery (Table 1) [2–4, 7, 40–42]. Notwithstanding that there may be physiological differences in the utilisation of substrates depending on the route and timing of nutrition delivery, strategies to enhance nutrition delivery [62, 63] should be factored into clinical trials where the outcome is dependent upon meeting a target.

### Duration of the intervention

Biological plausibility is fundamental to any nutrition research. This includes the likelihood that an outcome will be observed at a particular time point as a result of the specified duration of an intervention. Effects from nutrition dosing are unlikely to be observed immediately and any effect will be reliant on the consistency of delivery of the intervention. For example, the EDEN trial reported no difference in physical outcomes at 1 year when trophic or full feeding was given for 6 days [37]. Given that current data suggest that oral intake is inadequate post extubation [64, 65] and in the post-ICU recovery phase [66], it may be unlikely that an effect would be observed 1 year after such a short intervention. Observational studies tend to include patients fed over a longer period of time and exclude those patients fed over shorter durations. Because of the risk of bias associated with the short duration of trials, we propose that critical care nutrition studies should continue for longer than the first week of critical illness and should consider post-ICU nutritional intake.

## Outcomes

Whilst it is acknowledged that outcomes research is a priority for survivors of critical illness, no consensus exists on the most appropriate outcomes. There is considerable current activity in relation to core outcome sets in studies of physical rehabilitation [67] and long-term follow-up following acute respiratory failure [68] but no such initiative is ongoing for nutrition. Core outcome sets enable the combination and comparison of data from different studies of similar interventions and are urgently required in this field. Indeed, a recent scoping review of outcome measurement in ICU survivorship research from 1970–2013 found that 250 unique measurement instruments have been used across 425 studies [69]. Furthermore, only 31 RCTs included post-discharge outcomes and half of these had sample sizes of less than 100 subjects. Recent large trials undertaken in critical care nutrition have continued this pattern and utilised a variety of primary outcome measures from mortality to infectious complications and length of stay [2–4, 7, 40, 41, 70]. Whilst meta-analysis of such data is possible, the number of different outcome measures used profoundly limits the validity of any conclusions.

None of the large nutrition RCTs has used functional or health-related quality of life (HRQOL) measures as primary outcomes, but rather they have been included as secondary outcomes. This seems surprising, given that these are likely to be the outcomes where nutrition may show the most benefit [71], but reflects the current uncertainty regarding the most appropriate measure to use across the continuum of critical illness and recovery [14]. It is common that a significant number of patients are effectively excluded from recording of physical and functional ability due to either the volitional nature of the measure [72] or due to logistical issues with returning to follow-up appointments. Overall, these outcome measures can be labour intensive and expensive for the researcher, which may also impact on the choice of outcomes used for each study, the time points chosen to measure and the number of patients able to be followed up long term. Accounting for logistics and costs associated with measuring long-term outcomes is essential to the success of future trials.

## Recovery from critical illness

There are few studies investigating nutritional support after the first week of critical illness. However, oral intake has been reported to be inadequate in patients following extubation [64, 65], regardless of the presence of enteral feeding [65]. Failure to meet nutritional targets following the first week of ICU stay and into the post-ICU phase may indeed negatively influence any long-term measurements of skeletal muscle mass and physical or functional ability, and may be a confounder in studies measuring these outcomes in recent clinical trials. In addition, post-ICU studies which have included varying degrees of nutrition intervention have shown conflicting results [73, 74] and therefore little is understood about the clinical effectiveness of these interventions in the post-ICU phase However, it is clear that this is a research priority [75].

Furthermore, the investigation of multi-modal interventions, coupling appropriate nutrition and exercise interventions at specific time points, is warranted given the physiological evidence that increases in muscle mass and improvements in exercise capability are stronger when these interventions are provided in tandem [15]. Indeed, a study investigating the effects of a combined nutrition and exercise intervention in the ICU is due to commence later this year [76]. The outcome of extending the combination of these two interventions in the post-ICU phase should also be investigated.

## Conclusion

As survivorship after critical illness becomes an increasing focus of attention, future trials of nutrition during and following critical illness should consider specific factors that could provide measureable benefits in terms of both physical and functional recovery. Such factors include strategies to ensure adequate delivery of the intervention, provision of nutrition over a time frame in which it is biologically plausible to observe a difference in the desired outcome and selection of appropriate and consistent outcomes recorded at clinically relevant time points. Important outcomes include muscle mass, function and quality of life. In addition, patient selection of those most likely to benefit from nutritional interventions and nutrition research in the post-ICU phase merits specific attention.

## Abbreviations

ASPEN: American Society of Parenteral and Enteral Nutrition
CT: Computed tomography
EDEN: Early vs Delayed Enteral Feeding to Treat People with Acute Lung Injury or Acute Respiratory Distress Syndrome
EN: Enteral nutrition
EPaNIC: Early Parenteral Nutrition Completing Enteral Nutrition in Adult Critically Ill Patients
HRQOL: Health-related quality of life
ICU: Intensive care unit
MEE: Measured energy expenditure
MRC: Medical Research Council
MUSCLE UK: Musculoskeletal Ultrasound Study in Critical Care: Longitudinal Evaluation UK
NUTRIC: Nutrition Risk in the Critically Ill
PN: Parenteral nutrition
RCT: Randomised controlled trial
REDOXs: Reducing Deaths due to Oxidative Stress Study
SCCM: Society of Critical Care Medicine
SF-36: Short Form-36 health survey
